# Re-instatement of *Sorbus harrowiana* (Rosaceae), based on morphometric analysis

**DOI:** 10.3897/phytokeys.166.57672

**Published:** 2020-10-29

**Authors:** Meng Li, Steven P. Sylvester, Zhang-Pei Wang, Yi-Da Pei, Xin-Fen Gao, Yuan Zhao, Wen-Qi Jiang

**Affiliations:** 1 Co-Innovation Center for Sustainable Forestry in Southern China, College of Biology and the Environment, Nanjing Forestry University, Nanjing, 210037, China; 2 Chengdu Institute of Biology, Chinese Academy of Sciences, Chengdu, 610041, China; 3 College of Forestry, Nanjing Forestry University, Nanjing, 210037, China

**Keywords:** George Forrest, herbarium research, morphometric analysis, *
Pyrus
*, taxonomy

## Abstract

*Sorbus harrowiana* (≡*Pyrus harrowiana*), previously considered a synonym of *Sorbus insignis* (≡*Pyrus insignis*) in the *Flora of China*, is re-instated here and shown to be distinct from *S. insignis*, based on morphometric analysis, coupled with herbarium and field investigation. We also present for the first-time full descriptions, distributional records and notes for *S. harrowiana* and *S. insignis*.

## Introduction

*Sorbus* L. *sensu lato* (Rosaceae) contains approximately 260 species that widely occur in the temperate zone of the Northern Hemisphere (Yü and Lu 1974; Phipps et al. 1990; Aldasoro et al. 1998; Lu and Spongberg 2003). However, recent research using both molecular (Campbell et al. 2007; Lo and Donoghue 2012) and morphological evidence (Zheng and Zhang 2007) suggests that *Sorbussensu lato* is polyphyletic. *Sorbus* has been separated into six distinct genera: *Aria* (Pers.) Host, *Chamaemespilus* Medikus, *Micromeles* Decaisne, *Torminalis* Medikus, *Sorbussensu stricto* and *Cormus* Spach. *Sorbus* L. *sensu stricto* (Rosaceae) comprises ca. 80 species which are delimited from the aforementioned genera by their pinnately compound-leaves and are mainly distributed in the Northern Hemisphere: one or two species in Europe, seven species in North America and ca. 70 species throughout Asia (Hooker 1878; Warburg and Kárpáti 1968; Phipps et al. 1990; Lu and Spongberg 2003; McAllister 2005; Zika and Bailleul 2015).

Many species within *Sorbus* are difficult to delimit, including *S. insignis* (Hook. f.) Hedl. (≡*Pyrus insignis* Hook. f.), which bears similarities with *S. harrowiana* (Balf. f. & W. W. Smith) Rehd. (≡*Pyrus harrowiana* Balf. f. & W.W. Smith) in fruits and strong branchlets.

*Sorbus insignis* was first published by J. D. Hooker and C B. Clarke (Hooker 1878) in the genus *Pyrus* (≡*Pyrus insignis* Hook. f.), based on the collections from the Sikkim Himalaya. In the protologue, Hooker (1878) described *S. insignis* as a small, very robust tree with 4–6 paired leaflets per compound leaf and leaflet blades 3–4 inches (ca. 7–10 cm) long. Then, Hedlund (1901) transferred it to the genus *Sorbus*, based on its pinnately compound-leaves.

*Sorbus harrowiana* was described on a single George Forrest collection from the Yunnan, south-western China. In the protologue, Rehder (1920) emphasised the large leaflets which often exceed 8 inches (ca. 20 cm) in length and stated that *S. harrowiana* is markedly different from *S. insignis*, although the exact differences were not mentioned. Most taxonomists followed his opinion and considered the two to be distinct species (Yü and Lu 1974; Long 1987; Phipps et al. 1990; Kress et al. 2003; McAllister 2005; Watson and Manandhar 2012).

Gabrielian (1978), however, treated *S. harrowiana* as a synonym of *S. insignis* in the taxonomic and nomenclatural works of the genus *Sorbus* in western Asia and the Himalayas, but gave no detailed explanation. This treatment was subsequently adopted in the *Flora of China* (Lu and Spongberg 2003) and Tropicos (https://www.tropicos.org). During our taxonomic research on *Sorbus*, we found that these two species are easily identified and distinguishable in terms of morphological characteristics and geographical distribution. Herein, we evaluate the morphological variation between *S. insignis* and *S. harrowiana* through a morphometric study of herbarium specimens to establish the delimitation and validity of the two species and to produce a taxonomic treatment including descriptions and distributional notes.

## Materials and methods

### Plant material and characters scored

Herbarium specimens were studied at A, CAS, CDBI, GH, E, IBSC, K, KATH, KUN, LBG, MO, P, PE, TI and WU (abbreviations according to Thiers 2019). Leaflet length, width and length/width ratio (l/w ratio) were chosen as the morphometric variables to be measured amongst 133 specimen sheets (17 of *Sorbus insignis* and 116 of *S. harrowiana*) in 56 collections from across the range of their geographic distribution (Suppl. material 1: Table S1). Two to four compound leaves were randomly selected from each specimen. Two middle leaflets from each compound leaf were selected for measurement. A total of 1323 leaflets were measured (141 of *S. insignis* and 1128 of *S. harrowiana*).

### Statistical analyses

Normality of the QN standardised variables was tested using the Shapiro and Wilk test (Mahibbur and Govindarajulu 1997), with statistically-significant differences set by the p value < 0.05. Length and width data did not fit a normal distribution, even after log_10_- or square root- transformation, with only l/w data fitting a normal distribution after log_10_ transformation. As such, we performed analyses using both non-parametric Wilcoxon signed-rank tests on untransformed data and parametric Welch Two Sample T-tests on log_10_ transformed data as T-tests are considered robust to non-normality when large sample sizes are used. The analyses were performed in R version 4.0.2 (R Foundation for Statistical Computing, Vienna, AT). The data matrix for analysis was prepared in Microsoft Excel 2011 and is available on request.

## Results

### Morphometric analysis

**Figure 1.**
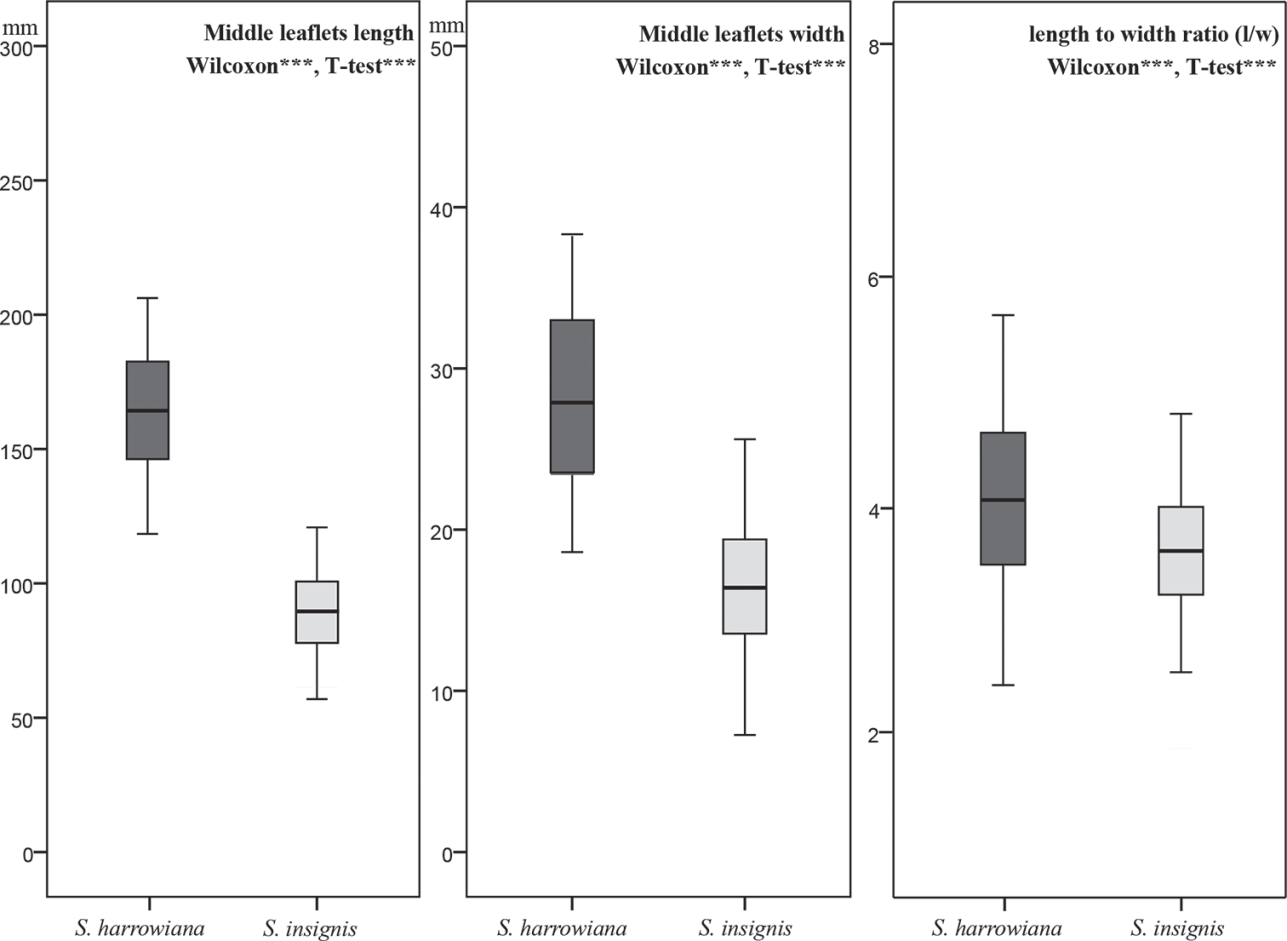
Box plot of comparisons between *S. harrowiana* and *S. insignis* in middle leaflet length, width and l/w ratio. Significant differences found upon analysis using non-parametric Wilcoxon rank sum test on untransformed data (Wilcoxon) and parametric Welch Two Sample T-test on log_10_ transformed data (T-test) are noted within the figures (*** = p < 0.001).

Both the non-parametric Wilcoxon signed-rank tests on untransformed data and parametric Welch Two Sample T-tests on log_10_ transformed data found highly significant (p < 0.001) differences between *S. harrowiana* and *S. insignis* in terms of middle leaflet length, width and l/w ratio (Fig. 1). Further detailed examination revealed other morphological characters, such as number and spacing of leaflets on the compound leaves and leaflet shape, venation and indumentum, that differentiate these two species: *Sorbus insignis* possesses pinnately compound leaves with 7–16 leaflets, leaflets at intervals of 1–2 cm, oblong or oblong-lanceolate, 5–8 × 1.5–2.0 cm, middle leaflets l/w ratio 3.3–4.0, lateral veins 24–30 pairs, both surfaces tomentose when young, glabrescent with age. *Sorbus harrowiana* has pinnately compound leaves with 5–7 leaflets, leaflets at intervals of 1–2.5 cm, oblong, 10–18 × 2.5–3.5 cm, middle leaflets l/w ratio 3.5–4.5, lateral veins 19–21 pairs, both surfaces glabrous. Our critical observations and morphometric analyses of herbarium specimens (including type pictures) demonstrate that *S. harrowiana* should be recognised as distinct from *S. insignis*.

### Taxonomic treatment

#### Sorbus insignis

Taxon classificationPlantaeRosalesRosaceae

(Hook. f.) Hedl., Kongl. Svenska Vetenskapsakad. Handl. 35(1): 32. 1901.

729682F3-3755-51E9-A4BC-6483196B2F92

≡Pyrus insignis Hook. f., Fl. Brit. India 2(5): 377. 1878.

##### Type.

**Figure 2.**
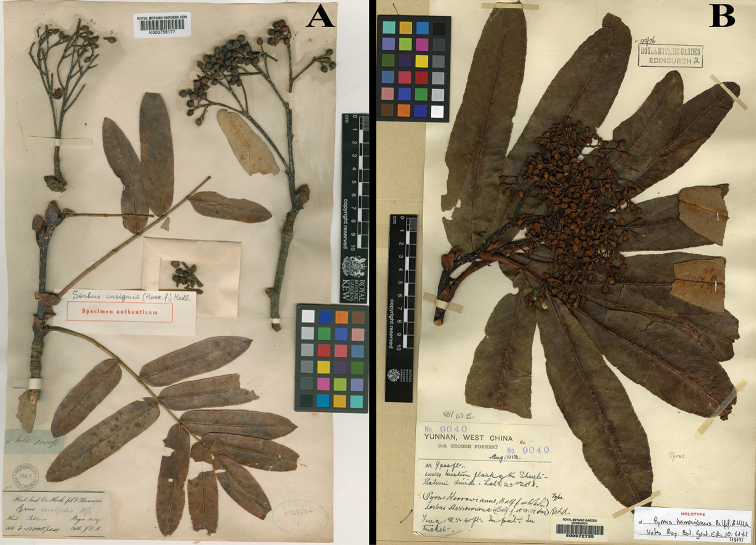
Type pictures of *Sorbus insignis* (Hook. f.) Hedl. (**A** barcode K000758177) and *S. harrowiana* (Balf. f. & W. W. Smith) Rehd. (**B** barcode E00072735).

India. **Sikkim**: 8000–11000 ft [2438–3353 m alt.], *J.D. Hooker & C.B. Clarke s.n.* (holotype: K (K000758177 [image!])). Fig. 2A.

##### Description.

Trees or shrubs. Stems greyish-brown; bark grey; branchlets greyish-brown when old; rusty brown tomentose when young, glabrous when old; winter buds narrowly ovoid, 1.0–1.8 cm long, scales initially puberulent, glabrate later; apex shortly acuminate or acute, brownish. Leaves pinnately compound, 15–20 cm long; stipules persistent, suborbicular, sometimes lobed, 1–2 cm wide, margin entire or serrate with pointed teeth; petiole 1.7–4.0 cm long, glabrous; rachis sparsely tomentose, glabrous when old; leaflets 7–16, at intervals of 1–2 cm long, oblong or oblong-lanceolate, 5–8 × 1.5–2.0 cm, l/w ratio 3.2–4.2, middle leaflets l/w ratio 3.3–4.0, lateral veins 24–30 pairs, margin slightly revolute and shallowly crenate, base rounded or obliquely cordate, apex obtuse, both surfaces tomentose when young, glabrescent. Inflorescences compound corymbs, 30–80 flowered, 8–12 cm in diam., peduncles sparsely pubescent; pedicels 2–4 mm long, sparsely pubescent; flowers 6–8 mm in diam.; hypanthium abaxially pubescent; sepals triangular, ca. 1.5 mm long, glabrous or somewhat puberulous, apex acute, with reddish dentate glands along margin; petals creamy white, suborbicular, ca. 4 × 3 mm; stamens ca. 20; carpels 3(–4); styles 2 or 3, 2.5 mm. Infructescence sparsely pubescent or glabrous; pomes green when young, white or crimson when mature, globose or ovoid-globose, 5–8 mm in diam., sepals persistent erect; seeds brownish, reniform, 1.5–2.0 mm long; 2n = 34.

##### Phenology.

Flowering May–June; fruiting Sep–Oct.

##### Distribution and habitat.

**Figure 3.**
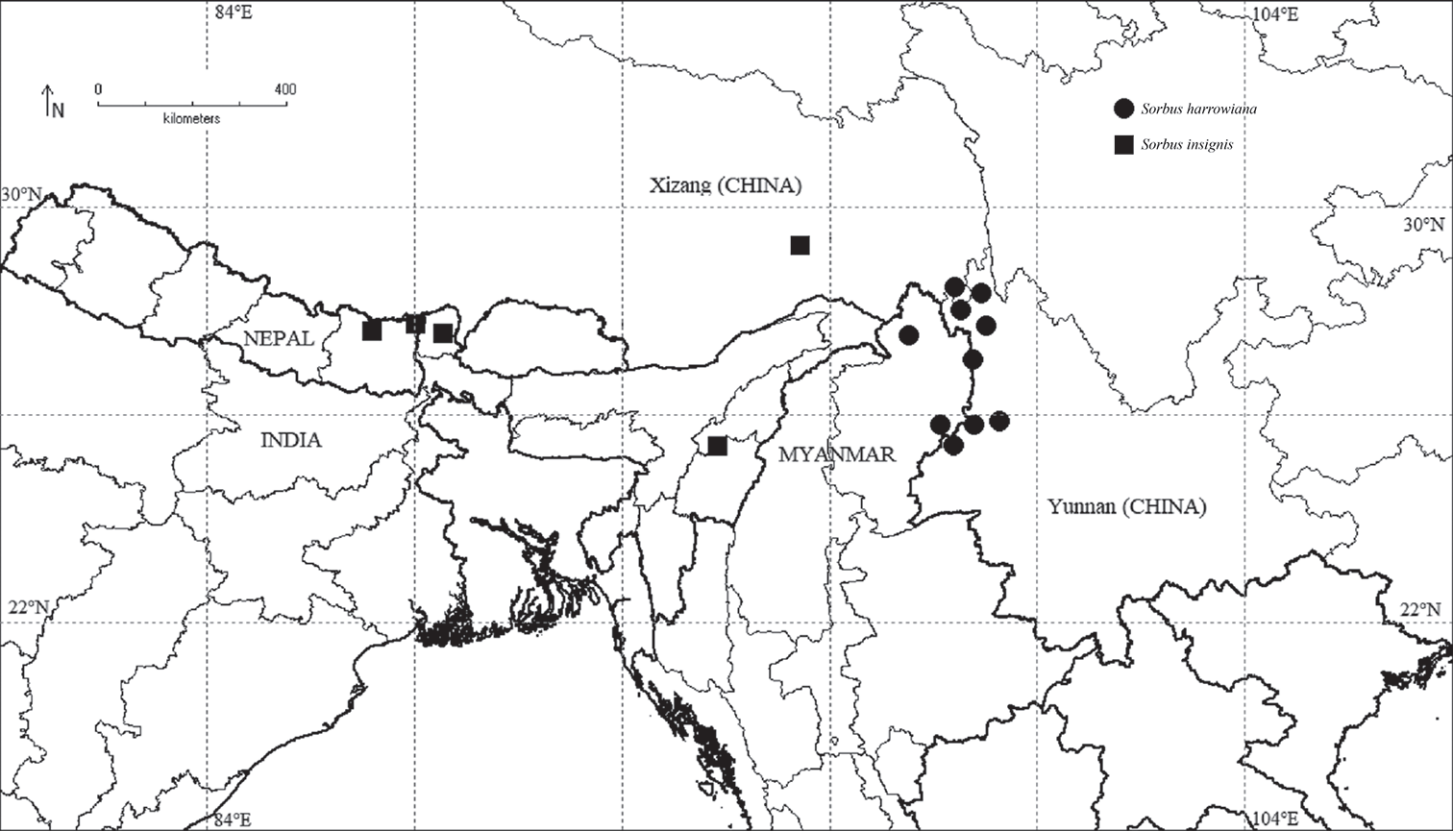
Distribution of *Sorbus insignis* and *S. harrowiana* at county level.

*Sorbus insignis* is distributed in India, Nepal and China (Xizang) (Fig. 3). It is also mentioned as occurring in ‘NW Yunnan’ China (Lu and Spongberg 2003), but we have not yet seen any convincing specimens from Yunnan. All specimens labelled as ‘*S. insignis*’ from Yunnan that we have examined were misidentified. It grows in broad-leaved forests on rocky slopes with an altitudinal range between 2500 and 3100 m.

##### Additional specimens examined.

**China**. **Xizang province**: Motuo, 2900 m alt., 25 Oct 2016, *B. Xu et al. YLZB4493* (CDBI); Galung La, 2700 m, 18 Aug 1982, *S.Z. Cheng & B.S. Li 0200* (PE).

**India**. **Manipur state**: Sirohifurar, 2500 m alt., Jan. 1982, *G. Watt 5965* (P); Ching Sow, 2400–2700 m alt., 16 April 1982, *G. Watt 6539* (P); **Sikkim state**: *T. Thomson s.n.* (E).

**Nepal**. **Eastern Nepal**: Sankhuwasabha, 29 Oct 1963, *H. Hara et al. 6301809* (TI); Kuwa Pani, 2890 m alt., 23 Aug 1997, *S. Noshiro et al. 9755184* (A, E); Kauma, 3130 m alt., 25 Sep 1991, *Edinburgh Makalu Expedition 259* (E, KATH); Koping, 3040 m alt., 24 Oct 1991, *Edinburgh Makalu Expedition 1019* (E); Jhakpu, 2750 m alt., 26 Sep 1971, *P.R. Shikyo & M. Ohsawa 1069* (KATH); Bhyaha, 2880 m alt., 31 Oct 1981, *P.R. Shakya 7296* (KATH); Taplejung, 2700 m alt., 30 Sep 1981, *P.R. Shakya 6777* (KATH).

#### Sorbus harrowiana

Taxon classificationPlantaeRosalesRosaceae

(Balf. f. & W. W. Smith) Rehd., J. Arnold Arbor. 1(4): 263. 1920.

6217B966-50F9-54F8-B8C3-96DC277C6406

≡Pyrus harrowiana Balf. f. & W.W. Smith, Notes Roy. Bot. Gard. Edinburgh 10 (46): 61–62. 1917.

##### Type.

China. **Yunnan**: Western flanks of the Shweli-Salwin Divide (Tenchong County?), 2800 m alt., 15 Aug 1912, *George Forrest 9040* (holotype: E (E00072735 [image!]); isotypes: A (A00046014!), E (E00010858 [image!], E00072736 [image!]), K (K000758123 [image!])). Fig. 2B.

##### Description.

Small trees or shrubs. Stems greyish-brown; bark grey to bronze; branchlets glabrous; winter buds conic, 1.5–2.0 cm long, scales glabrous; apex acute, reddish-brown. Leaves pinnately compound, 15–25 cm long; stipules persistent, suborbicular, 1–2 cm, margin toothed; petiole 2.0–4.0 cm long, glabrous; rachis sparsely tomentose, glabrous when old; leaflets 5–7, at intervals of 1–2.5 cm long, oblong, 10–18 × 2.5–3.5 cm, l/w ratio 3.2–4.6, middle leaflets l/w ratio 3.5–4.5, lateral veins 19–21 pairs, margin slightly revolute and shallowly crenate, base rounded or obliquely cordate, apex rounded, both surfaces glabrous. Inflorescences compound corymbs, 30–200 flowered, 12–20 cm in diam., peduncles sparsely pubescent or glabrous; pedicels 3–6 mm, sparsely pubescent or glabrous; flowers 6–9 mm in diam.; hypanthium abaxially pubescent; sepals triangular, ca. 2.0 mm long, glabrous or somewhat puberulous, apex acute, with serrate along margin; petals creamy white, suborbicular, ca. 4 × 4 mm; stamens ca. 15–25; carpels 3(–4); styles 2–4, 2–4 mm. Infructescences sparsely pubescent or glabrous; pomes creamy-white rarely pink, globose or ovoid-globose, 5–7 mm in diam., sepals persistent erect; seeds brownish, reniform, 1.5–2.0 mm long; 2n = 34.

##### Phenology.

Flowering May–July; fruiting Sep–Oct.

##### Distribution and habitat.

**Figure 4.**
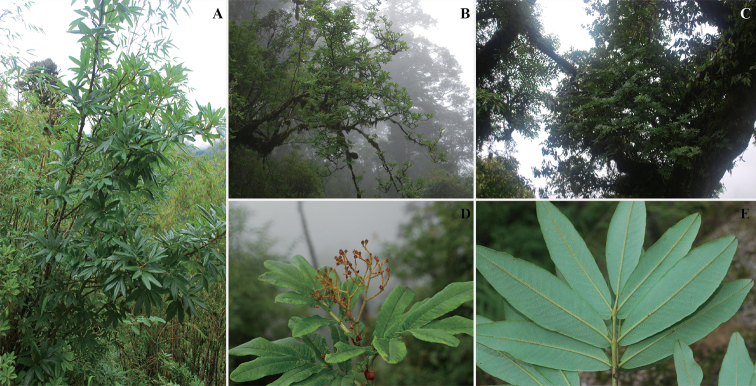
*Sorbus harrowiana***A** growing near the road in mixed forest **B** growing on a steep forested slope above road **C** growing on *Lithorcarpus* like an epiphytic shrub **D** fruiting branch **E** pinnately compound-leaves.

*Sorbus harrowiana* is distributed in China and Myanmar (Fig. 3). It grows in broad-leaved forests with an altitudinal range between 2100 and 3300 m. (Fig. 4).

##### Additional specimens examined.

**China**. **Yunnan province**: Deqin, Cizhong, 2800 m alt., 8 Oct 1959, *K.M. Feng 23997* (KUN, PE); Fugong, Lumadeng Xiang, 3100 m alt., 13 Aug 2005, *Gaoligong Shan Biodiversity Survey* (*G.S.B.S.*) *27252* (CAS, GH, KUN); Biluo Mountain, 2800 m alt., 28 May 1982, *Qinghai-Tibet Expedition 6939* (KUN, PE); Yaping pass, 2884 m alt., 2 May 2002, *G.S.B.S. 20209* (CAS, E, GH, KUN, MO); Yaduo Cun, 2700 m alt., 6 Aug 2005, *G.S.B.S. 26500* (CAS, GH, KUN); Shibali, 2700 m alt., 15 Aug 2005, *G.S.B.S. 28304* (CAS, GH, KUN); *G.S.B.S. 28409* (CAS, GH, KUN); Gongshan, Bingzhongluo, 2530 m alt., 1 Sep 2006, *G.S.B.S. 31826* (CAS, E, GH, MO, KUN); 2700 m alt., 25 Jul 1982, *Qinghai-Tibet Expedition 7512* (KUN, PE); Zguziluo, 2800–3000 m alt., 25 Jun 1978, *Bijiang Expedition 1009* (KUN); Champutung, 3000 m alt., Oct 1935, *C.W. Wang 67581* (A, KUN, LBG, NAS, PE, PE, WUK); Dulongjiang, 2700 m alt., 21 May 1991, *Dulongjiang Expedition 7022* (KUN); Kongdang, 3030 m alt., 28 Sep 2002, *G.S.B.S. 16655* (CAS, E, GH, KUN); Labadi, 2970 m alt., 30 Sep 2002, *G.S.B.S. 16792* (CAS, E, GH, KUN); Pula river, 3100 m alt., 3 Oct 2002, *G.S.B.S. 16906* (CAS, E, GH, KUN); Fucai, 2780 m alt., 1 Sep 2006, *G.S.B.S. 31772* (CAS, E, GH, MO, KUN); Yidongwei, 2530 m alt., 1 Sep 2006, *G.S.B.S. 31826* (CAS, E, GH, MO, KUN); Mangzhou Wadi, 3010 m alt., 12 Aug 2006, *G.S.B.S. 33217* (CAS, E, G, KUN); Qi-Qi Nature Reserve Station, 3200 m alt., 16 Oct 1996, *G.S.B.S.* (*1996*) *7803* (E); Dong Shao Fang, 3100 m alt., 21 Sep 1997, *G.S.B.S.* (*1997*) *9495* (E, MO); Danzhu river, 2750 m alt., 1 Jul 2000, *H. Li et al. 11855* (CAS, E GH, KUN); Dulong Jiang valley, 2770–3050 m alt., 15 Jul 2000, *H. Li et al. 12614* (CAS, E, GH, MO, KUN); Su Ki Tung, 1923, *J. Rock 10161* (A); Mt. Kenyichunpo, 10000 ft [3048 m alt.], Jul 1923, *J. Rock 22068* (A, E); Cikai, 07 Oct 1940, *K.M. Feng 7946* (KUN, PE); East Check Point, 2700 m alt., 22 Jul 1982, *K.M. Feng 8226* (KUN, PE); Wangtzang, 2800 m alt., 13 Sep 1938, *T.T. Yü 20216* (A, E, KUN, PE); Swangchiang, 13 Jul 1938, *T.T. Yü 22088* (A, E, KUN, PE); Lushui, 2800 m alt., 6 Jul 2016, *Meng Li et al. GYD0058* (CDBI); Tenchong, Mingguang Xiang, 19 May 2006, 3000–3050 m alt., *G.S.B.S. 29157* (CAS, E, GH, KUN); Zizhi Cun, 2750–2850 m alt., 19 May 2006, *G.S.B.S. 29224* (CAS, E, GH, MO, KUN); Jiangao Shan, 3020 m alt., 23 May 2006, *G.S.B.S. 30546* (CAS, E, GH, MO, KUN); Weixi, 2900 m alt., 13 Oct 1934, *H.T. Tsai 59790* (A, IBSC, KUN, P, PE); 2800 m alt., 20 Oct 1934, *H.T. Tsai 59902* (A, IBSC, KUN, LBG, PE); Biluo Snow Mountain, 3250 m alt., 11 Jul 1981, *PE Hengduan Mountain Collection Team 01402* (PE); Cangjiang river, 3300 m alt., 18 May 1940, *K.M. Feng 3965* (KUN, PE); Baimaluo, 3200 m alt., 3 June 1940, *K.M. Feng 4429* (KUN, PE); Yanwa, 3600 m alt., 11 May 1960, *Kunming Workstation of Institute of Botany 8375* (KUN, PE); Kangpucheda, 3000 m alt., 8 Oct 1956, *P.I. Mao 385* (KUN, PE); Magelo, 23 Aug 1956, *P.I. Mao 401* (KUN, PE); Bading, 3500 m alt., 11 May 1982, *Qinghai-Tibet Expedition 6434* (PE); Yunlong, Nu Shan, 2 Nov. 1993, *K.D. Rushforth 2663* (E); **Xizang province**: Chayu, 10000–11000 ft [3048–3353 m alt.], June 1922, *G. Forrest 21806* (A, E, P); Oct. 1922, *G. Forrest 22870* (A, E).

**Myanmar**. **Kachin state**: Myitkyina, 3050 m alt., 19 Oct 1919, *R. Farrer 1403* (E); *G. Forrest 24481* (E, PE); Chimili, *G. Forrest 26813* (A, E); *G. Forrest 27541* (A, E); *G. Forrest* 29775 (E, PE); Putao, 2800 m alt., 16 May 1953, *F. Kingdon-Ward 20921* (E); Tata Bum, 3000 m alt., 19 June 1953, *F. Kingdon-Ward 21009* (A, E); Trails above Camp 3, 3000–3140 m alt., 22 Oct 2013, *Hponyin Mountains Expedition 206A* (E); Adung velly, *F. Kingdon-Ward 9568* (A).

### Key to species in Sorbus Ser. Insignes Yü.

**Note.** George Forrest made seven major expeditions to western China and north-eastern Myanmar during 1917 and 1931, with over 30,000 collections being made. In these expeditions, G. Forrest collected at least 23 specimens of *Sorbus harrowiana*, of which 15 lacked locality information. However, from limited collection information, we are able to broadly georeference these 15 specimens to four different regions (as he mentioned in the collection record), i.e. (1) China: Yunnan: Shweli-Salwin divide, *G. Forrest 15888* (E, P), *G. Forrest 16101* (E), *G. Forrest 18506* (E), *G. Forrest 24373* (E, P, PE), *G. Forrest 26778* (A, E); (2) China: Yunnan: N’mai kha-Salwin divide, *G. Forrest 18527* (E, P), *G. Forrest 18753* (E); (3) China: Yunnan: Salwin-Kui Chiang divide, *G. Forrest 25687* (E, PE), *G. Forrest 25768* (E); (4) China: Yunnan, *G. Forrest 19874* (E), *G. Forrest 20849* (E), *G. Forrest 20869* (E, P), *G. Forrest 21806* (A, E, P), *G. Forrest 29015* (E, PE), *G. Forrest 30374* (E, PE). The specimens examined by us also covered those four regions.

**Table:** 

1	Leaflets, Inflorescences and infructescence pubescent	***S. helenae* Koehne**
–	Leaflets, inflorescences and infructescence glabrous	**2**
2	Leaflets 7–16 per compound leaf, 5–8 × 1.5–2.0 cm, oblong or oblong-lanceolate, lateral veins 24–30 pairs, both surfaces tomentose when young, glabrescent with age	***S. insignis***
–	Leaflets 5–7 per compound leaf, 10–18 × 2.5–3.5 cm, oblong, lateral veins 19–21 pairs, both surfaces glabrous	***S. harrowiana***

## Supplementary Material

XML Treatment for Sorbus insignis

XML Treatment for Sorbus harrowiana

## Supplementary materials

Supplementary material 1 Table S1. Accessions of herbarium specimen for measurements Data type measurement Explanation note . https://binary.pensoft.net/file/469011This dataset is made available under the Open Database License (http://opendatacommons.org/licenses/odbl/1.0/). The Open Database License (ODbL) is a license agreement intended to allow users to freely share, modify, and use this Dataset while maintaining this same freedom for others, provided that the original source and author(s) are credited.Meng Li, Steven P. Sylvester, Zhang-Pei Wang, Yi-Da Pei, Xin-Fen Gao, Yuan Zhao, Wen-Qi Jiang

## References

[B1] Aldasoro JJ Aedo C Navarro C Garmendia FM (1998) The genus *Sorbus* (Maloideae, Rosaceae) in Europe and in North Africa: Morphological analysis and systematics. Systematic Botany 23(2): 189–212. 10.2307/2419588

[B2] Campbell CS Evans RC Morgan DR Dickinson TA Arsenault MP (2007) Phylogeny of subtribe Pyrinae (formerly the Maloideae, Rosaceae): limited resolution of a complex evolutionary history. Plant Systematics and Evolution 266: 119–145. 10.1007/s00606-007-0545-y

[B3] Gabrielian EC (1978) The Genus *Sorbus* in Western Asia and the Himalayas. Izdatielstwo Akademii Nauk Armianskoj SSR, Erevan, 263 pp. [In Russian]

[B4] Hedlund JT (1901) Monographie der Gattung *Sorbus*. Kongliga Svenska Vetenskaps-Akademiens Handlingar 35: 1–147.

[B5] Hooker JD (1878) Rosaceae. In: Hooker JD (Ed.) The Flora of British India (Vol.2.). L. Reeve, London, 307–388.

[B6] Kress WJ DeFilipps RA Farr E Kyi DYY (2003) A checklist of the trees, shrubs, herbs, and climbers of Myanmar (Revised from the original works by J. H. Lace, R. Rodger, H. G. Hundley, and U Chit Ko Ko on the “List of trees, shrubs, herbs and principal climbers, etc. recorded from Burma”). Contributions from the U.S. National Herbarium, Washington, 590 pp.

[B7] Lo EYY Donoghue MJ (2012) Expanded phylogenetic and dating analyses of the apples and their relatives (Pyreae, Rosaceae). Molecular Phylogenetics and Evolution 63(2): 230–243. 10.1016/j.ympev.2011.10.005 22293154

[B8] Long DG (1987) *Sorbus*. In: Grierson AJC Long DG (Eds) Flora of Bhutan: Including a Record of Plants from Sikkim (Vol.1, Part 3). Royal Botanic Garden Edinburgh, Edinburg, 592–599.

[B9] Lu LT Spongberg SA (2003) *Sorbus* L. In: Wu Z-Y Raven PH Hong D-Y (Eds) Flora of China (Vol.9). Science Press, Beijing & Missouri Botanical Garden Press, Saint Louis, 144–170.

[B10] Mahibbur MR Govindarajulu Z (1997) A modification of the test of Shapiro and Wilk for normality. Journal of Applied Statistics 24(2): 219–236. 10.1080/02664769723828

[B11] McAllister H (2005) The Genus *Sorbus*-Mountain Ash and Other Rowans. Royal Botanic Gardens, Kew, 252 pp.

[B12] Phipps JB Robertson KR Smith PG Rohrer JR (1990) A checklist of the subfamily Maloideae (Rosaceae). Canadian Journal of Botany 68(10): 2209–2269. 10.1139/b90-288

[B13] Rehder A (1920) New species, varieties and combinations from the Herbarium and collections of the Arnold Arboretum. Journal of the Arnold Arboretum 1: 256–264. 10.5962/bhl.part.17092

[B14] Thiers B (2019) Index Herbariorum: A global directory of public herbaria and associated staff. New York Botanical Garden’s virtual herbarium. The New York Botanical Garden. [accessed 05.2019] http://sweetgum.nybg.org/ih/

[B15] Warburg EF Kárpáti ZE (1968) *Sorbus* L. In: Tutin TG Heywood VH Burges NA Moore DM Valentine DH Walters SM Webb DA (Eds) Flora Europaea (Vol.2). Rosaceae to Umbelliferae. Cambridge University Press, Cambridge, 67–71.

[B16] Watson MF Manandhar VK (2012) *Sorbus* L. In: Watson MF Akiyama S Ikeda H Pendry CA Rajbhandari KR Shrestha KK (Eds) Flora of Nepal.Royal Botanic Garden Edinburgh, Edinburgh, 25–32.

[B17] Yü TT Lu LT (1974) *Sorbus* L. In: Yü TT (Ed.) Flora Reipublicae Popularis Sinicae (Vol.36). Science Press, Beijing, 283–344. [In Chinese]

[B18] Zheng DM Zhang ML (2007) A Cladistic and Phenetic Analysis of the Infrageneric Relationships of Sorbus s.l. (Maloideae, Rosaceae) Based on the Morphological Characters. Acta Horticulturae Sinica 34: 723–728. [In Chinese] 10.3321/j.issn:0513-353x.2007.03.033

[B19] Zika PF Bailleul SM (2015) *Sorbus* L. In: FNA Ed Committee (Ed.) Flora of North America, North of Mexico (Vol.9). Oxford University Press, New York, 433–445.

