# Supplementary material for: Re-instatement of Sorbus harrowiana (Rosaceae), based on morphometric analysis
Source: PhytoKeys. 2020 Oct 29;166:29–39. doi: 10.3897/phytokeys.166.57672 (PMC7644610; doi:10.3897/phytokeys.166.57672)
Supplement: Supplementary material 1 — Table S1. Accessions of herbarium specimen for measurements [file phytokeys-166-029-s001.docx]

Table S1. Accessions of herbarium specimen for measurements

| **Scientific Name** | **Collector** | **Collector No.** | **Location** | **Date** | **Barcode / ID** | **Herbarium** |
| --- | --- | --- | --- | --- | --- | --- |
| *Sorbus harrowiana* | F. Kingdon-Ward | 9568 | MYANMAR. Kachin. Putao | 1937/5/31 | N/A | A |
| *S. harrowiana* | Hponyin Mountains Expedition | 206A | MYANMAR. Kachin. Putao | 2013/10/22 | E00871719 | E |
| *S. harrowiana* | R. Farrer | 1403 | MYANMAR. Kachin. Myitkyina | 1919/10/19 | E00072796 | E |
| *S. harrowiana* | G. Forrest | 27541 | MYANMAR. Kachin. Myitkyina | 1925/10/1 | N/A | A |
| *S. harrowiana* | G. Forrest | 27541 | MYANMAR. Kachin. Myitkyina | 1925/10/1 | E00072797 | E |
| *S. harrowiana* | K.D. Rushforth | 2663 | CHINA. Yunan. Yunlong | 1993/11/2 | E00073615 | E |
| *S. harrowiana* | H.T. Tsai | 59790 | CHINA. Yunan. Weixi | 1934/10/13 | A00138530 | A |
| *S. harrowiana* | H.T. Tsai | 59790 | CHINA. Yunan. Weixi | 1934/10/13 | IBSC0343961 | IBSC |
| *S. harrowiana* | H.T. Tsai | 59790 | CHINA. Yunan. Weixi | 1934/10/13 | KUN0667638 | KUN |
| *S. harrowiana* | H.T. Tsai | 59790 | CHINA. Yunan. Weixi | 1934/10/13 | P03240323 | P |
| *S. harrowiana* | H.T. Tsai | 59790 | CHINA. Yunan. Weixi | 1934/10/13 | PE00962389 | PE |
| *S. harrowiana* | H.T. Tsai | 59902 | CHINA. Yunan. Weixi | 1934/10/20 | A00138532 | A |
| *S. harrowiana* | H.T. Tsai | 59902 | CHINA. Yunan. Weixi | 1934/10/20 | KUN0667639 | KUN |
| *S. harrowiana* | H.T. Tsai | 59902 | CHINA. Yunan. Weixi | 1934/10/20 | LBG00073596 | LBG |
| *S. harrowiana* | H.T. Tsai | 59902 | CHINA. Yunan. Weixi | 1934/10/20 | PE00962387 | PE |
| *S. harrowiana* | P.I. Mao | 401 | CHINA. Yunan. Weixi | 1956/8/23 | KUN0667641 | KUN |
| *S. harrowiana* | P.I. Mao | 401 | CHINA. Yunan. Weixi | 1956/8/23 | PE00962386 | PE |
| *S. harrowiana* | P.I. Mao | 385 | CHINA. Yunan. Weixi | 1956/10/8 | KUN0667640 | KUN |
| *S. harrowiana* | P.I. Mao | 385 | CHINA. Yunan. Weixi | 1956/10/8 | PE00962385 | PE |
| *S. harrowiana* | G. Forrest | 9040 | CHINA. Yunan. Tengchong | 1912/8/15 | E00010858 | E |
| *S. harrowiana* | G. Forrest | 9040 | CHINA. Yunan. Tengchong | 1912/8/15 | E00072735 | E |
| *S. harrowiana* | G. Forrest | 9040 | CHINA. Yunan. Tengchong | 1912/8/15 | E00072736 | E |
| *S. harrowiana* | G. Forrest | 9040 | CHINA. Yunan. Tengchong | 1912/8/15 | GH00046017 | GH |
| *S. harrowiana* | G. Forrest | 9040 | CHINA. Yunan. Tengchong | 1912/8/15 | K000758123 | K |
| *S. harrowiana* | C.W. Wang | 67581 | CHINA. Yunan. Gongshan | 1935/10/1 | A00138536 | A |
| *S. harrowiana* | C.W. Wang | 67581 | CHINA. Yunan. Gongshan | 1935/10/1 | KUN0667685 | KUN |
| *S. harrowiana* | C.W. Wang | 67581 | CHINA. Yunan. Gongshan | 1935/10/1 | LBG00073570 | LBG |
| *S. harrowiana* | C.W. Wang | 67581 | CHINA. Yunan. Gongshan | 1935/10/1 | PE00962370 | PE |
| *S. harrowiana* | C.W. Wang | 67581 | CHINA. Yunan. Gongshan | 1935/10/1 | PE00962371 | PE |
| *S. harrowiana* | T.T. Yü | 22088 | CHINA. Yunan. Gongshan | 1938/7/13 | E00072738 | E |
| *S. harrowiana* | T.T. Yü | 22088 | CHINA. Yunan. Gongshan | 1938/7/13 | KUN0667691 | KUN |
| *S. harrowiana* | T.T. Yü | 22088 | CHINA. Yunan. Gongshan | 1938/7/13 | KUN0667692 | KUN |
| *S. harrowiana* | T.T. Yü | 22088 | CHINA. Yunan. Gongshan | 1938/7/13 | PE00962372 | PE |
| *S. harrowiana* | T.T. Yü | 22088 | CHINA. Yunan. Gongshan | 1938/7/13 | PE00962373 | PE |
| *S. harrowiana* | T.T. Yü | 20216 | CHINA. Yunan. Gongshan | 1938/9/13 | A00138538 | A |
| *S. harrowiana* | T.T. Yü | 20216 | CHINA. Yunan. Gongshan | 1938/9/13 | E00072737 | E |
| *S. harrowiana* | T.T. Yü | 20216 | CHINA. Yunan. Gongshan | 1938/9/13 | KUN0667693 | KUN |
| *S. harrowiana* | T.T. Yü | 20216 | CHINA. Yunan. Gongshan | 1938/9/13 | PE00962379 | PE |
| *S. harrowiana* | T.T. Yü | 22088 | CHINA. Yunan. Gongshan | 1938/9/13 | A00138535 | A |
| *S. harrowiana* | K.M. Feng | 7946 | CHINA. Yunan. Gongshan | 1940/10/7 | KUN0667694 | KUN |
| *S. harrowiana* | K.M. Feng | 7946 | CHINA. Yunan. Gongshan | 1940/10/7 | KUN0667695 | KUN |
| *S. harrowiana* | K.M. Feng | 7946 | CHINA. Yunan. Gongshan | 1940/10/7 | PE00962374 | PE |
| *S. harrowiana* | K.M. Feng | 7946 | CHINA. Yunan. Gongshan | 1940/10/7 | PE00962375 | PE |
| *S. harrowiana* | Bijiang Expedition | 1009 | CHINA. Yunan. Gongshan | 1978/7/11 | KUN667689 | KUN |
| *S. harrowiana* | Bijiang Expedition | 1009 | CHINA. Yunan. Gongshan | 1978/7/11 | KUN667690 | KUN |
| *S. harrowiana* | Qinghai-Tibet Expedition | 7512 | CHINA. Yunan. Gongshan | 1982/6/25 | KUN0667683 | KUN |
| *S. harrowiana* | Qinghai-Tibet Expedition | 7512 | CHINA. Yunan. Gongshan | 1982/6/25 | KUN0667684 | KUN |
| *S. harrowiana* | Qinghai-Tibet Expedition | 7512 | CHINA. Yunan. Gongshan | 1982/6/25 | PE01159863 | PE |
| *S. harrowiana* | Qinghai-Tibet Expedition | 7512 | CHINA. Yunan. Gongshan | 1982/6/25 | PE01159864 | PE |
| *S. harrowiana* | Qinghai-Tibet Expedition | 7512 | CHINA. Yunan. Gongshan | 1982/6/25 | PE01612387 | PE |
| *S. harrowiana* | K.M. Feng | 8226 | CHINA. Yunan. Gongshan | 1982/7/22 | KUN0667696 | KUN |
| *S. harrowiana* | K.M. Feng | 8226 | CHINA. Yunan. Gongshan | 1982/7/22 | KUN0667697 | KUN |
| *S. harrowiana* | K.M. Feng | 8226 | CHINA. Yunan. Gongshan | 1982/7/22 | PE00962376 | PE |
| *S. harrowiana* | K.M. Feng | 8226 | CHINA. Yunan. Gongshan | 1982/7/22 | PE00962377 | PE |
| *S. harrowiana* | Qinghai-Tibet Expedition | 8421 | CHINA. Yunan. Gongshan | 1982/7/22 | KUN0667679 | KUN |
| *S. harrowiana* | Qinghai-Tibet Expedition | 8421 | CHINA. Yunan. Gongshan | 1982/7/22 | KUN0667680 | KUN |
| *S. harrowiana* | Qinghai-Tibet Expedition | 8421 | CHINA. Yunan. Gongshan | 1982/7/22 | PE01159860 | PE |
| *S. harrowiana* | Qinghai-Tibet Expedition | 8421 | CHINA. Yunan. Gongshan | 1982/7/22 | PE01159861 | PE |
| *S. harrowiana* | Qinghai-Tibet Expedition | 8421 | CHINA. Yunan. Gongshan | 1982/7/22 | PE01159862 | PE |
| *S. harrowiana* | Gaoligong Shan Biodiversity Survey | 7803 | CHINA. Yunan. Gongshan | 1996/10/16 | E00092412 | E |
| *S. harrowiana* | Gaoligong Shan Biodiversity Survey | 7803 | CHINA. Yunan. Gongshan | 1996/10/16 | E00746990 | E |
| *S. harrowiana* | Gaoligong Shan Biodiversity Survey | 7803 | CHINA. Yunan. Gongshan | 1996/10/16 | E00747002 | E |
| *S. harrowiana* | Gaoligong Shan Biodiversity Survey | 9495 | CHINA. Yunan. Gongshan | 1997/9/21 | E00092402 | E |
| *S. harrowiana* | Gaoligong Shan Biodiversity Survey | 9495 | CHINA. Yunan. Gongshan | 1997/9/21 | MO5190640 | MO |
| *S. harrowiana* | Heng Li et al. | 11855 | CHINA. Yunan. Gongshan | 2000/7/1 | E00416529 | E |
| *S. harrowiana* | Heng Li et al. | 11855 | CHINA. Yunan. Gongshan | 2000/7/1 | GH00138528 | GH |
| *S. harrowiana* | Heng Li et al. | 12614 | CHINA. Yunan. Gongshan | 2000/7/15 | E00416533 | E |
| *S. harrowiana* | Heng Li et al. | 12614 | CHINA. Yunan. Gongshan | 2000/7/15 | GH00138527 | GH |
| *S. harrowiana* | Heng Li et al. | 12614 | CHINA. Yunan. Gongshan | 2000/7/15 | MO5754017 | MO |
| *S. harrowiana* | Gaoligong Shan Biodiversity Survey | 16655 | CHINA. Yunan. Gongshan | 2002/9/28 | E00913102 | E |
| *S. harrowiana* | Gaoligong Shan Biodiversity Survey | 16655 | CHINA. Yunan. Gongshan | 2002/9/28 | GH00352334 | GH |
| *S. harrowiana* | Gaoligong Shan Biodiversity Survey | 16792 | CHINA. Yunan. Gongshan | 2002/9/30 | E00913104 | E |
| *S. harrowiana* | Gaoligong Shan Biodiversity Survey | 16792 | CHINA. Yunan. Gongshan | 2002/9/30 | GH00352295 | GH |
| *S. harrowiana* | Gaoligong Shan Biodiversity Survey | 16906 | CHINA. Yunan. Gongshan | 2002/10/3 | E00913106 | E |
| *S. harrowiana* | Gaoligong Shan Biodiversity Survey | 16906 | CHINA. Yunan. Gongshan | 2002/10/3 | GH00352306 | GH |
| *S. harrowiana* | Gaoligong Shan Biodiversity Survey | 16906 | CHINA. Yunan. Gongshan | 2002/10/3 | KUN1415514 | KUN |
| *S. harrowiana* | Gaoligong Shan Biodiversity Survey | 16906 | CHINA. Yunan. Gongshan | 2002/10/3 | KUN1415515 | KUN |
| *S. harrowiana* | Gaoligong Shan Biodiversity Survey | 16906 | CHINA. Yunan. Gongshan | 2002/10/3 | KUN1415516 | KUN |
| *S. harrowiana* | Gaoligong Shan Biodiversity Survey | 16906 | CHINA. Yunan. Gongshan | 2002/10/3 | KUN1415517 | KUN |
| *S. harrowiana* | Gaoligong Shan Biodiversity Survey | 16906 | CHINA. Yunan. Gongshan | 2002/10/3 | KUN1415518 | KUN |
| *S. harrowiana* | Gaoligong Shan Biodiversity Survey | 16906 | CHINA. Yunan. Gongshan | 2002/10/3 | KUN1415519 | KUN |
| *S. harrowiana* | Gaoligong Shan Biodiversity Survey | 16906 | CHINA. Yunan. Gongshan | 2002/10/3 | KUN1415520 | KUN |
| *S. harrowiana* | Gaoligong Shan Biodiversity Survey | 16906 | CHINA. Yunan. Gongshan | 2002/10/3 | KUN1415521 | KUN |
| *S. harrowiana* | Gaoligong Shan Biodiversity Survey | 33217 | CHINA. Yunan. Gongshan | 2006/8/12 | E00657388 | E |
| *S. harrowiana* | Gaoligong Shan Biodiversity Survey | 33217 | CHINA. Yunan. Gongshan | 2006/8/12 | GH00297183 | GH |
| *S. harrowiana* | Gaoligong Shan Biodiversity Survey | 31772 | CHINA. Yunan. Gongshan | 2006/9/1 | E00509768 | E |
| *S. harrowiana* | Gaoligong Shan Biodiversity Survey | 31772 | CHINA. Yunan. Gongshan | 2006/9/1 | GH00268908 | GH |
| *S. harrowiana* | Gaoligong Shan Biodiversity Survey | 31772 | CHINA. Yunan. Gongshan | 2006/9/1 | MO6051954 | MO |
| *S. harrowiana* | Gaoligong Shan Biodiversity Survey | 31826 | CHINA. Yunan. Gongshan | 2006/9/1 | CAS0325681 | CAS |
| *S. harrowiana* | Gaoligong Shan Biodiversity Survey | 31826 | CHINA. Yunan. Gongshan | 2006/9/1 | E00657293 | E |
| *S. harrowiana* | Gaoligong Shan Biodiversity Survey | 31826 | CHINA. Yunan. Gongshan | 2006/9/1 | GH00272546 | GH |
| *S. harrowiana* | Gaoligong Shan Biodiversity Survey | 31826 | CHINA. Yunan. Gongshan | 2006/9/1 | MO6056511 | MO |
| *S. harrowiana* | Gaoligong Shan Biodiversity Survey | 26500 | CHINA. Yunan. Fugong | 2005/8/6 | GH00237521 | GH |
| *S. harrowiana* | Gaoligong Shan Biodiversity Survey | 27252 | CHINA. Yunan. Fugong | 2005/8/13 | GH00266226 | GH |
| *S. harrowiana* | Gaoligong Shan Biodiversity Survey | 28304 | CHINA. Yunan. Fugong | 2005/8/15 | GH00266227 | GH |
| *S. harrowiana* | Gaoligong Shan Biodiversity Survey | 28409 | CHINA. Yunan. Fugong | 2005/8/15 | GH01176147 | GH |
| *S. harrowiana* | Qinghai-Tibet Expedition | 6939 | CHINA. Yunan. Fugong | 1982/5/28 | KUN0605907 | KUN |
| *S. harrowiana* | Qinghai-Tibet Expedition | 6939 | CHINA. Yunan. Fugong | 1982/5/28 | PE01159869 | PE |
| *S. harrowiana* | Qinghai-Tibet Expedition | 6939 | CHINA. Yunan. Fugong | 1982/5/28 | PE01159870 | PE |
| *S. harrowiana* | Qinghai-Tibet Expedition | 6939 | CHINA. Yunan. Fugong | 1982/5/28 | PE01596471 | PE |
| *S. harrowiana* | K.M. Feng | 23997 | CHINA. Yunan. Deqin | 1959/10/8 | KUN0667633 | KUN |
| *S. harrowiana* | K.M. Feng | 23997 | CHINA. Yunan. Deqin | 1959/10/8 | PE01596470 | PE |
| *S. harrowiana* | G. Forrest | 16101 | CHINA. Yunan | 1917/10/1 | E00622351 | E |
| *S. harrowiana* | G. Forrest | 18527 | CHINA. Yunan | 1923/ | P03240311 | P |
| *S. harrowiana* | G. Forrest | 20869 | CHINA. Yunan | 1923/ | P03240310 | P |
| *S. harrowiana* | G. Forrest | 24373 | CHINA. Yunan | 1924/6/18 | E00072744 | E |
| *S. harrowiana* | G. Forrest | 24373 | CHINA. Yunan | 1924/6/18 | P03240324 | P |
| *S. harrowiana* | G. Forrest | 24373 | CHINA. Yunan | 1924/6/18 | PE00962383 | PE |
| *S. harrowiana* | G. Forrest | 25768 | CHINA. Yunan | 1924/10/1 | E00622349 | E |
| *S. harrowiana* | G. Forrest | 29015 | CHINA. Yunan | 1930/ | E00622347 | E |
| *S. harrowiana* | G. Forrest | 29015 | CHINA. Yunan | 1930/ | PE01596472 | PE |
| *S. harrowiana* | G. Forrest | 29775 | CHINA. Yunan | 1931/6/ | PE01596469 | PE |
| *S. harrowiana* | G. Forrest | 30374 | CHINA. Yunan | unknown | E00622348 | E |
| *S. harrowiana* | G. Forrest | 30374 | CHINA. Yunan | unknown | PE01596468 | PE |
| *S. harrowiana* | G. Forrest | 21806 | CHINA. Xizang. Chayu | 1922/6/ | A00138529 | A |
| *S. harrowiana* | G. Forrest | 21806 | CHINA. Xizang. Chayu | 1922/6/ | P03240309 | P |
| *S. insignis* | P.R. Shakya | 6777 | NEPAL. Eastern Nepal. Taplejung | 2020/9/30 | N/A | KATH |
| *S. insignis* | H. Hara et al. | 6301809 | NEPAL. Eastern Nepal. Sankhuwasabha | 1963/10/29 | TI | TI |
| *S. insignis* | P.R. Shikyo & M. Ohsawa | 1069 | NEPAL. Eastern Nepal. Sankhuwasabha | 1971/9/26 | N/A | KATH |
| *S. insignis* | P.R. Shakya | 7296 | NEPAL. Eastern Nepal. Sankhuwasabha | 1981/10/31 | N/A | KATH |
| *S. insignis* | P.R. Shakya | 7296 | NEPAL. Eastern Nepal. Sankhuwasabha | 1981/10/31 | N/A | KATH |
| *S. insignis* | Edinburgh Makalu Expedition | 259 | NEPAL. Eastern Nepal. Sankhuwasabha | 1991/9/25 | E00262254 | E |
| *S. insignis* | Edinburgh Makalu Expedition | 259 | NEPAL. Eastern Nepal. Sankhuwasabha | 1991/9/25 | KATH010596 | KATH |
| *S. insignis* | Edinburgh Makalu Expedition | 1019 | NEPAL. Eastern Nepal. Sankhuwasabha | 1991/10/24 | E00262255 | E |
| *S. insignis* | S. Noshiro et al. | 9755184 | NEPAL. Eastern Nepal. Sankhuwasabha | 1997/8/23 | N/A | A |
| *S. insignis* | S. Noshiro et al. | 9755184 | NEPAL. Eastern Nepal. Sankhuwasabha | 1997/8/23 | E00224502 | E |
| *S. insignis* | S. Noshiro et al. | 9755184 | NEPAL. Eastern Nepal. Sankhuwasabha | 1997/8/23 | E00224503 | E |
| *S. insignis* | T. Thomson | s.n. | INDIA. Sikkim | unknown | E00262256 | E |
| *S. insignis* | J.D. Hooker & C.B. Clarke | s.n. | INDIA. Sikkim | unknown | K000758177 | K |
| *S. insignis* | G. Watt | 5965 | INDIA. Manipur. Sirohifurar | 1882/1/ | P03240325 | P |
| *S. insignis* | G. Watt | 6539 | INDIA. Manipur. Sirohifurar | 1882/4/16 | P03240326 | P |
| *S. insignis* | S.Z. Cheng & B.S. Li | 200 | CHINA. Xizang. Motuo | 1982/8/18 | PE01612393 | PE |
| *S. insignis* | S.Z. Cheng & B.S. Li | 200 | CHINA. Xizang. Motuo | 1982/8/18 | PE01612394 | PE |
